# Exosomal miR-452-5p Induce M2 Macrophage Polarization to Accelerate Hepatocellular Carcinoma Progression by Targeting TIMP3

**DOI:** 10.1155/2022/1032106

**Published:** 2022-09-16

**Authors:** Hu Zongqiang, Chen Jiapeng, Zhao Yingpeng, Yan Chuntao, Wang Yiting, Zhu Jiashun, Li Li

**Affiliations:** ^1^Department of Hepato-Pancreato-Biliary Surgery, First People's Hospital of Kunming, No. 504 Qinnian Road, Kunming, 650032 Yunnan, China; ^2^Department of Hepato-Pancreato-Biliary Surgery, The Calmette Affiliated Hospital of Kunming Medical University, Kunming, 650032 Yunnan, China

## Abstract

**Background:**

Hepatocellular carcinoma (HCC) cell-derived exosomes have shown effects on inducing M2 macrophage polarization and promoting HCC progression. MiR-452-5p was reported by recent studies to promote malignancy progression as an exosomal microRNA that secreted by HCC cells, of which the underlying mechanism remains unclear. Here, we further explored how miR-452-5p functions in HCC.

**Methods:**

MiR-452-5p expressions in HCC cells was examined by in situ hybridization. Next, HCC cell lines were transfected with the mimics or the inhibitor of miR-452-5p. Transfected cells' biological behavior were analyzed by CCK-8, flow cytometry, and Transwell assay. Then, exosomes were purified from miR-452-5p inhibited or overexpressed HCC cells and cocultured with macrophages to examine the role of miR-452-5p in macrophage polarization. To examine the role of exosomal miR-452-5p on macrophage polarization and tumor growth. We also performed the dual-luciferase assay to explore the targeting relationship between miR-452-5p and TIMP3.

**Results:**

The upregulation of miR-452-5p was identified in HCC. The effects of HCC cell-derived exosomes on accelerating HCC migration and invasion and inducing M2 macrophage polarization were confirmed, which were further enhanced after overexpressing miR-452-5p but neutralized after silencing miR-452-5p. In addition, *in vivo* experiments demonstrated the effect of miR-452-5p on accelerating HCC growth and metastasis. Also, we identified that TIMP3 overexpression inhibited the promoted cell invasion and migration by HCC cell-derived exosomes.

**Conclusion:**

Exosomal miR-452-5p secreted from HCC cells could induce polarization of M2 macrophage and therefore stimulating HCC progression by targeting TIMP3. Thus, miR-452-5p might be a potential biomarker for HCC prognosis.

## 1. Introduction

Hepatocellular carcinoma (HCC) is one of the primary causes for cancer death worldwide, especially in Asian areas [1]. Although efforts and improvements have been made in HCC treatment during past decades, HCC patients are still suffered from poor prognosis, high frequency of tumor recurrence, and metastasis, resulting in a low 5-year survival rate [2, 3]. HCC diagnoses are usually processed without histological studies due to the risk of tumor seeding for invasive biopsies, which makes HCC prognosis by noninvasive biopsy such important [4]. In recent years, biological markers have been increasingly studied for HCC diagnosis and prognosis, but it still remains a long way to go to fully elucidate the molecular mechanisms. Therefore, it is of significance to seek promising biomarkers for early HCC diagnosis.

MicroRNAs are small, conserved noncoding RNAs that can modulate gene via suppressing protein expression or inducing their target mRNAs degradation at the posttranscriptional stage. Their dysregulation has been revealed to be related with tumor progression [5, 6], some of which play essential roles in HCC development and metastasis and serve as suppressors or oncogenes, contributing to HCC early diagnosis [7]. Exosomes have recently been revealed as carriers of chemokines, growth factors, and miRNAs for promoting intercellular communication and tumorigenesis [8, 9]. They are small extracellular vesicles spread from various cells especially tumor cells and their phospholipid bilayer structures help to protect the carrying miRNAs from degradation by RNase in the body [10]. Exosomes secreted by tumor cells are 10 times more than that from normal cells, and studies on tumor-derived exosomal miRNAs have been increasingly conducted these years [11]. Additionally, exosomal miRNAs have been demonstrated to show similar functions as miRNAs in cells of origin, such as affecting tumor cells invasion and metastasis as well as retaining tumor growth [12], suggesting their promising value for cancer diagnosis.

Previous study by Wang et al. detected more stable and higher expression of miR-21 in serum exosome than that of HCC serum [13]. By detecting the expression of exosome miRNAs in serum of patients with liver cirrhosis and HCC, Sohn et al. found that the expression of exosome miRNAs in the serum of patients with HCC was significantly different from that of patients with other diseases [14]. All these findings provide us an idea that exosomal miRNAs might be valuable for HCC prognosis. As an oncogene, miR-452-5p has been reported to exert impacts on several malignancies [15–17]. And a previous study indicated that miR-452-5p mediated the proliferation, migration, and invasion of HCC [15]. However, whether and how exome-derived miR-452-5p functions in HCC remains undisclosed. Therefore, in this study, we explored the role of exosomal miR-452-5p in HCC progression.

More and more attention has been paid to the relationship between tumor cells and their surrounding microenvironment. Tumor-associated macrophages (TAMs) as a sort of inflammatory cells are able to accelerate cancer progression and metastasis, and a strong relationship between poor survival rate and high macrophage density has been found in HCC [16, 17]. Normally, macrophages are polarized into two different types of macrophages, called M1 or M2 macrophages, in which M2-polarized macrophages constitute most of TAMs and exert primary functions. Studies in liver diseases have reported that tumor-released exosomes can be transferred to or ingested by TAMs, resulting in the acceleration of tumor metastasis [18]. In addition, exosomes generated from HCC cells have been identified to promote M2 macrophage polarization and facilitate immune escape of tumor cells [19]. Considering that the functions of exosomes from M2 macrophages remain enigmatic, it is worth exploring the effect of M2 macrophage exosomes on the biological behavior of HCC cells and its molecular mechanism.

Thus, we aimed to explore how exosomal miR-452-5p affected HCC cell progression and whether exosomal miR-452-5p could affect macrophage polarization. In addition, the human tissue inhibitor of metalloprotease 3 (TIMP3), which acted as an inhibitory gene in HCC [20], was proved as a miR-452-5p target. By understanding the underlying mechanisms of exosomal miR-452-5p/TIMP3 during HCC development, we might provide novel views for HCC diagnosis.

## 2. Materials and Methods

### 2.1. Online Database

We verified the expression of miR-452-5p in HCC tissues by the TCGA database (https://www.cancer.gov/types/liver). The overall survival (OS), disease-free survival (DFS), and ROC curves were further analyzed by R software (ver. 3.6.3). miRNA-mRNA targeting prediction was performed using starBase (https://starbase.sysu.edu.cn/agoClipRNA.php?source=mRNA). GO enrichment analysis was achieved by using Enrichr (https://maayanlab.cloud/Enrichr/).

### 2.2. Human Tissue Samples

Five pairs of hepatocellular carcinoma (HCC) tissues and paracancer tissues involved in this study were provided by the First People's Hospital of Kunming City. All patients involved have not received other therapeutics before operation. This study was carried out after the approval of the Research ethics Committee of Kunming Medical University. The informed consent was signed by every involved patient.

### 2.3. Cell Culture and Transfection

HCC cells (Huh-7, MHCC97-L, HCCLM3, and SNU-182), normal human epithelial cell THLE-3, and THP-1 monocytes were obtained from ATCC. Cells were grown in DMEM (Thermo Fisher scientific) supplemented with 1% penicillin/streptomycin (Gibco) and 10% FBS (Gibco), incubated at 37°C with 5% CO_2_. THP-1 cells were incubated in 150 ng/mL of PMA for 1-2 days.

For cell transfection, miR-452-5p inhibitor, and mimics and their relative controls, the TIMP3 overexpression vector and the empty vector were obtained from GenePharma. Transfection of cells was completed by referring to Lipofectamine 2000 (Invitrogen) kit. After 48 hours incubation, cells were isolated and ready for the following experiments.

### 2.4. In Situ Hybridization (ISH)

The miR-452-5p expression level was detected by using a specific miR-452-5p probe (Boster, Wuhan, China). Sections were deparaffinized and incubated with probes for 24 h at 40°C. After that, sections were stained with hematoxylin and dehydrated in graded ethanol and xylene. Then, the DAB substrate was added.

### 2.5. Real-Time Quantitative PCR (qRT-PCR)

Extraction of total RNA by the TRIzol method, miRNAs were extracted by using miRNeasy Mini Kit (Qiagen, Germany) followed by quantitative RT-PCR analysis. cDNA was synthesized from 1 *μ*g RNA using PrimeScript RT reagent. qRT-PCR detection was achieved by using SYBR premix Ex Taq II kit (ELK bioscience) on an ABI 7300 RT-PCR system. Expression was normalized with GAPDH and U6 served as the controls and analyzed by 2^-*ΔΔ*CT^ method. All primer sequences in Table S1.

### 2.6. Cell Proliferation and Apoptosis Assay

To determine the viability of cells, CCK-8 solution (10 *μ*L/well) were added into cells in the 96-well plates. After one-hour incubation, cell absorbance was measured at 450 nm.

Cells proliferation was analyzed by EdU assay. In brief, cells were incubated with EdU assay kit (Ribobio) for 2 hours at 37°C and then incubated with DAPI at room temperature for 5 min. Finally, cells were mount and imaged under a fluorescence microscope.

Cell apoptosis was determined by flow cytometry. Cells were washed for 3 times in PBS, gently resuspended in binding solution, and then treated in 5 *μ*L of Annexin V-FITC/PI for 10 minutes at 4°C in dark. All the data was analyzed by a flow cytometer (BD Biosciences).

### 2.7. Transwell Invasion and Migration Assay

Firstly, precoat the upper chamber with 100 *μ*L Matrigel (only involved in the transwell invasion assay). Subsequently, the fused monolayer cells (5 × 10^4^/mL) were centrifuged and resuspended with serum-free DMEM before being transferred to the upper chamber. Meanwhile, 10% DMEM was added into the lower compartment. The plate was incubated for 24-48 hours at 37°C. After the nonmigrating or noninvading cells in the upper chamber were exfoliated, the cells migrated to the lower surface of the membrane were first fixed and then stained with crystal violet. Finally, the number of cells was counted under a microscope.

### 2.8. Exosome Purification and RNase Treatment

Once the cells achieved a confluent monolayer, they were centrifuged and washed with PBS and transferred into exosome-free medium with 10% FBS. The exosomes from cell culture were extracted by gradient centrifugation. Subsequently, the supernatant was ultracentrifuged in 100,000 × *g* for an hour. The morphology of isolated exosomes was observed under TEM and photographed, and then, western blot was used to detect exosomes markers.

### 2.9. Transmission Electron Microscopy (TEM)

In preparation for photographing exosomes, the exosome pellets were fixed in PBS/2.5% glutaraldehyde (pH 7.2) for an hour and washed with PBS. The exosomes were then fixed with 1% osmium tetroxide for 1 hour at 4°C. The fixed exosomes were embedded in 10% gelatin and cut into blocks. Exosomes are dehydrated by graded alcohol and transferred to propylene oxide, which is then infiltrated with graded Quetol-812 epoxy resin, polymerized overnight at 37°C, and then incubated for 24 hr at 60°C. The resulting specimens were cut into sections of 60 nm, then stained with 2% uranyl acetate for 30 minutes, and then stained with citric acid for 15 minutes. Sections were photographed under a FEI Tecnai T20 TEM at 80 KV.

### 2.10. Western Blot

Total cell protein was extracted after cell lysis in RIPA buffer. The extracted proteins were segregated in 12% SDS-PAGE for 45 min and then coated onto the PVDF membrane. Incubate with 5% skim milk for at least 1 hour to avoid nonspecific binding and then incubate with primary antibody overnight at 4°C. After that, secondary antibody (1/2000, Invitrogen) was incubated for 1 hour at room temperature. Enhanced chemiluminescence display of immune response bands and counted by ImageJ software. Antibody information was listed in Table S2.

### 2.11. Immunofluorescence

Cells were fixed in methanol for half an hour and then incubated with anti-PKH26 (Abcam) overnight at 4°C. After cells were incubated with IgG (1/5000, Abcam) for an hour at room temperature. The staining was visualized under a fluorescence microscope.

### 2.12. Luciferase Reporter Assay

Construction of luciferase wild-type and mutant reporters (TIMP3-WT and TIMP3-MUT), and NC mimics and miR-452-5p mimics were cotransfected in THP-1 cells by Lipofectamine 2000 (Invitrogen). Luciferase activity was detected after 48 hours incubation.

### 2.13. Tumor Xenograft Animal Models

The experimental animals were 6-week-old male BALB/c nude mice, all of which were purchased from Slac Laboratory Animal Company, Shanghai. MHCC97-L cells (2 × 10^6^) cocultured with macrophages, macrophages^inhibitorNC-Huh7-exo^, or macrophages^miRinhibitor-Huh-7-exo^ were injected subcutaneously into mice. Tumors in the mice were measured weekly for 3 weeks; then, tumor xenografts were harvested after the mice were sacrificed. Animal experiments were approved by the Animal Care and Use Committee of the First People's Hospital of Kunming.

### 2.14. Statistical Analysis

The data was analyzed by Student's *t*-test (normally), while the nonnormally ones were analyzed by the unpaired Mann–Whitney test. The curves and rates of disease-free survival (DFS) and overall survival (OS) were analyzed by the Kaplan-Meier method, and the differences between groups were compared by log-rank test. GraphPad Prism 9 was applied for statistical analyses. When *P* value < 0.05, the difference was considered statistically significant.

## 3. Results

### 3.1. Diagnostic and Prognostic Values of miR-452-5p in HCC

**Figure 1:**
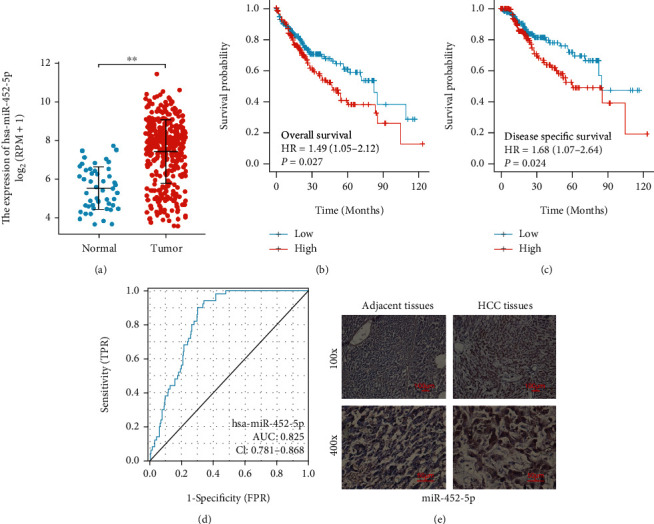
miR-452-5p is promising for HCC diagnosis and prognosis. (a) Scatter plots show relative miR-452-5p level in healthy and HCC samples accessed. (b, c) Overall and disease-free survival analyses. (d) ROC analysis. (e) ISH staining images of miR-452-5p expression. ^∗∗^*P* < 0.01. Scale bar: 100 *μ*m.

Based on the TCGA-LUAD data, the miR-452-5p expression was upregulated in tumor samples compared to the normal controls (Figure 1(a)). We compared the OS and DFS values of TCGA-LUAD patients with high or low expression of miR-452-5p to determine the prognostic value of miR-452-5p in HCC. The results showed that the expression of miR-452-5p was correlated with the survival rate of HCC patients, and the survival rate of HCC patients with high expression of miR-452-5p was poorer than that of HCC patients with low expression (Figures 1(b) and 1(c)). Additionally, a ROC was created with an AUC value of 0.825 (95% CI, 0.781-0.868, Figure 1(d)), and therefore, the diagnostic value of miR-452-5p was confirmed. Moreover, miR-452-5p was detected in tissue specimens by in situ hybridization assay. The staining results also confirmed a higher amount of miR-452-5p (Figure 1(e)). Therefore, these results demonstrated that a higher miR-452-5p expression in HCC.

### 3.2. miR-452-5p Inhibition Represses Cell Growth and Promotes Apoptosis in HCC Cells

**Figure 2:**
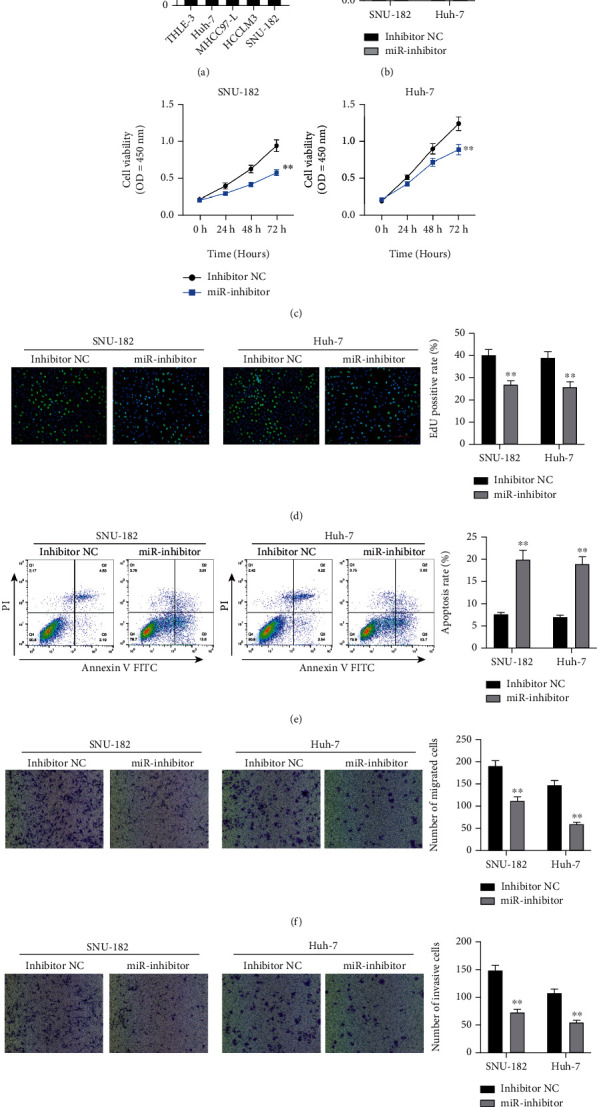
miR-452-5p inhibition suppressed HCC cell migration and invasion. (a) miR-452-5p expression in HCC cells and normal human epithelial cells. (b) miR-452-5p was successfully inhibited by the miR-inhibitor. (c, d) CCK-8 assay and EdU staining of HCC cells with and without miR-452-5p inhibition. Optical density (OD) was measured at 24, 48, and 72 h after transfection. (e) Apoptosis rate was analyzed by flow cytometry. (f, g) Migration and invasion were detected by Transwell assay. ^∗∗^*P* < 0.01.

We found that miR-452-5p expression was significantly upregulated in HCC cells compared with normal human epithelial cells (Figure 2(a)). Considering more meaningful results, SNU-182 and Huh-7 cells were selected to be used in subsequent experiments.

We firstly suppressed miR-452-5p expression in both Huh-7 and SNU-182 cells to see if the proliferation of those cells would be inhibited.  Figure 2(b) showed a successful transfection with significantly lower miR-452-5p expression in miR-inhibitor groups. CCK-8 assay and EdU staining were performed. As expected, we observed a significant decrease of cell growth after inhibiting miR-452-5p (Figures 2(c) and 2(d)). Moreover, flow cytometry showed that miR-452-5p inhibition could increase the apoptosis of cells significantly (Figure 2(e)), and a remarkably reduced migrated and invasive cells was observed in Transwell assay (Figures 2(f) and 2(g)).

We next wanted to know if the overexpression of miR-452-5p could show opposite effects on HCC cells, where miR-mimics was applied in cell transfection (Figure S1A). As we expected, miR-452-5p overexpression promoted cells growth (Figure S1B-C) but suppressed the apoptosis of HCC cells (Figure S1D) remarkably. In addition, there were significantly more migrated and invasive cells observed in miR-452-5p overexpressed HCC cells (Figure S1F-G).

### 3.3. miR-452-5p Resides in Exosomes Derived from HCC Cells

The systemic changes of tumor cells are mainly stimulated by the secretion of exosomes, in which a plenty of miRNAs are encapsulated and playing necessary roles in biological functions [21]. Thus, we hypothesized that exosomes might also be involved in the stimulation of HCC cells, which will be verified by analyzing miR-452-5p expression in HCC cell-derived exosomes or not.

**Figure 3:**
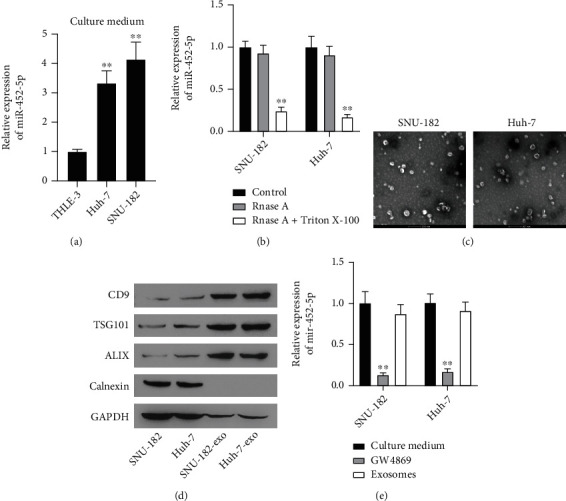
miR-452-5p mainly reside in HCC cells-derived exosomes. (a) miR-452-5p in the culture medium of normal epithelial cells and HCC cells. (b) miR-452-5p were encapsulated protected from RNase. (c, d) TEM and WB validation of purified exosomes from SNU-182 and Huh-7 cells. (e) miR-452-5p in HCC cells treated with GW4869 or purified exosomes are analyzed by qRT-PCR. ^∗∗^*P* < 0.01. Scale bar: 200 nm.

Cell culture mediums of THLE-3, Huh-7, and SNU-182 were collected for experiments, and significantly higher level of miR-452-5p was detected in HCC cells culture mediums than that from normal epithelial cells (Figure 3(a)). We also compared the level of miR-452-5p in HCC cells treated by RNase A with or without Triton X-100. We found that RNase A treatment showed little effect on miR-452-5p level, but the additional treatment of Triton X-100 reduced miR-452-5p level significantly (Figure 3(b)), suggesting that miR-452-5p were encapsulated and protected against RNase A. Therefore, it was hypothesized that miR-452-5p selectively resided in the exosome lumen derived by HCC cells. Purified exosomes were prepared by centrifugation and observed by transmission electron microscope (Figure 3(c)). Exosome protein markers were also detected by western blot. Compared to the negative control, CD9, TSG101, and ALIX were all remarkably expressed while Calnexin were not detected in SNU-182-exo and Huh-7-exo, suggesting the successful extraction and purification of exosomes (Figure 3(d)). To confirm if the HCC cell-derived exosomes are the source producing miR-452-5p, GW4869 was used to inhibit the secretion of HCC-derived exosomes. The results showed a remarkably decreased miR-452-5p expression in GW4869-treated HCC cells (Figure 3(e)), which was consistent with our expectation.

### 3.4. Exosomal miR-452-5p Induces M2 Macrophage Polarization

**Figure 4:**
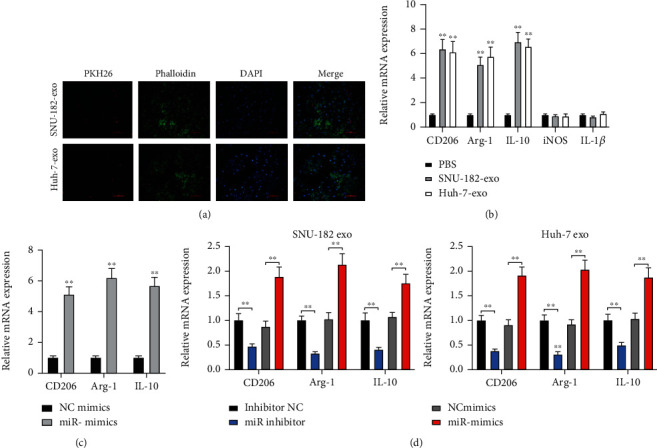
HCC cells deserved exosomal miR-452-5p induces M2 polarization of macrophages. (a) PKH26-labelled SNU-182-exo and Huh-7-exo. (b) mRNA expression of macrophage markers after coculturing with HCC cell exosome. (c) mRNA expression of M2 macrophage markers. (d) mRNA expression of M2 macrophage markers in macrophages treated with exosomes from miR-452-5p inhibited or overexpressed HCC cells. ^∗∗^*P* < 0.01. Scale bar: 100 *μ*m.

M2 macrophages contribute to the growth, invasion, and metastasis of tumor cells. To investigate if exosomes released from HCC cells could induce M2 macrophage polarization, PMA can promote the differentiation of THP-1 monocytes into M0 macrophages. We observed that PMA-induced THP-1 cells grew from single rounded cells into adherent cells (Figure S2A). In addition, after PMA-induced THP-1 cells, we detected a significant increase in the expression of the macrophage marker CD68 (Figure S2B), suggesting a successful cell induction. Next, the roles of HCC cells-derived exosomes on macrophage polarization were investigated. SNU-182-exo and Huh-7-exo were labelled by PKH26. After incubation for 48 hours with macrophages, positive PKH26 staining was observed, suggesting the exosomes were internalized by macrophages (Figure 4(a)). Moreover, the expression levels of CD206, IL-10, and Arg-1 (M2 macrophage makers) showed significantly increase while the expression of iNOS and IL-1*β* (M1 macrophage markers) observed no difference after coculturing with SNU-182-exo and Huh-7-exo (Figure 4(b)).

Subsequently, we inspected if miR-452-5p participated in the process of M2 macrophage polarization. In Figure 4(c), miR-452-5p overexpression resulted in an upregulated of CD206, IL-10, and Arg-1, suggesting that miR-452-5p could promoted the M2 macrophage polarization. Then, exosomes from the cells transfected with miR-inhibitor, miR-mimics, or negative controls were cocultured with macrophages (Figure 4(d)). M2 macrophage markers were significantly decreased in miR-inhibited exosomes and increased in miR-overexpressed exosomes.

### 3.5. Exosomal miR-452-5p Derived from HCC Cells Promotes Cell Migration, Invasion, and Tumorigenesis

**Figure 5:**
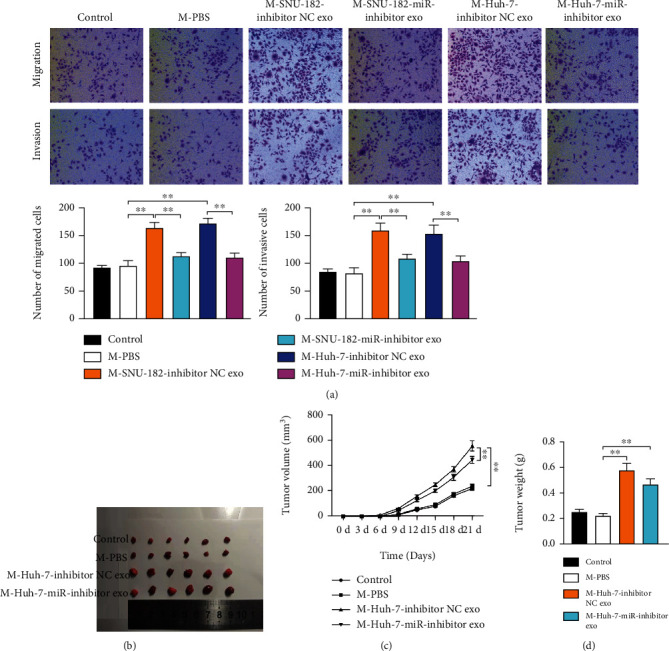
HCC cells deserved exosomal miR-452-5p accelerates M2 macrophage polarization to stimulate HCC cell migration, invasion *in vitro*, and tumorigenesis *in vivo*. (a) Migration and invasion rates of transfected MHCC97-L cells, M-PBS set up as a negative control. (b) Tumorigenicity of xenograft mice models. (c) Tumor volumes were measured each week for three weeks. (d) Tumor weights were measured after mice were sacrificed. ^∗∗^*P* < 0.01.

We next explored whether exosomal miR-452-5p accelerated HCC tumor progression. Macrophages were treated by SNU-182-exo or Huh-7-exo transferring miR-inhibitor or inhibitor NC and cocultured with HCC cell line MHCC97-L. By performing Transwell assay, we found that macrophages treated by HCC-cell-exo transferring miR-inhibitor functioned to normalise the increased cell migration and invasion rates (Figure 5(a)).

To demonstrate the tumorigenic ability of miR-452-5p *in vivo*, subcutaneous murine xenograft models were created and the xenograft tumor was measured once a week for three weeks. We found that the mice in the M-Huh-7-miR-inhibitor exo group had smaller tumor size compared to the mice in the inhibitor NC exo group (Figures 5(b)–5(d)). Therefore, we confirmed that HCC cells deserved exosomal miR-452-5p could accelerate HCC cells invasion and migration and tumorigenesis.

### 3.6. miR-452-5p Targets TIMP3 to Induce M2 Macrophage Polarization

**Figure 6:**
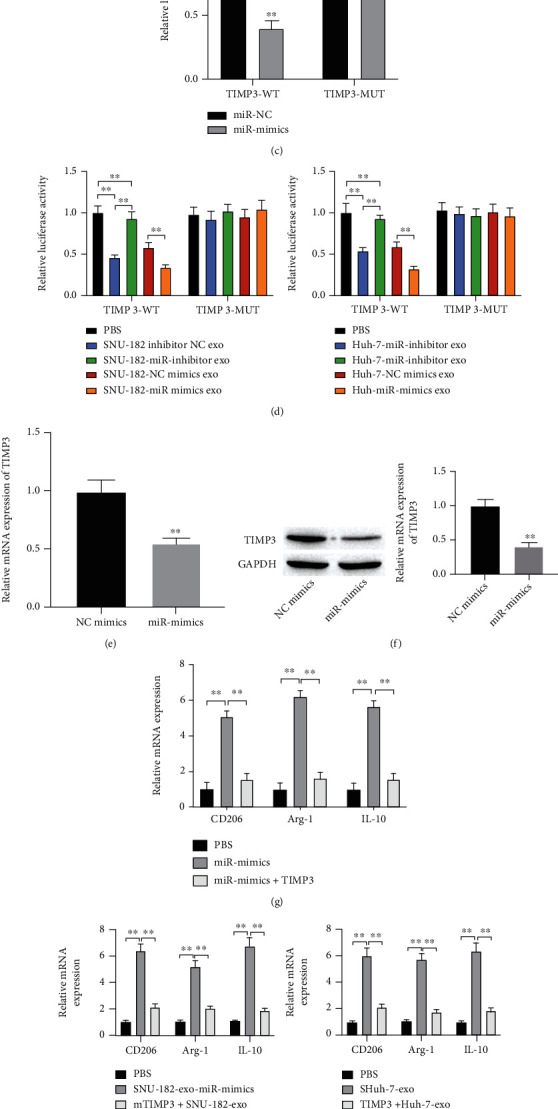
TIMP3 is a target of miR-452-5p in HCC. (a) Venn plot showed the overlapped genes predicted by starBase and TargetScan webtools. (b) Predicted binding sequence of miR-452-5p on TIMP3. (c) A dual-luciferase reporter assay. (d) A dual-luciferase reporter assay was performed to determine the effect of exosomal miR-492-5p on the luciferase activity. (e, f) The expression of both TIMP3 mRNA and protein decreased after overexpressing miR-452-5p in THP-1 cells. (g) mRNA expression of M2 macrophage markers. (h) mRNA expression of M2 macrophage markers in THP-1 cells after different treatment. (i) Cell migration and invasion detected by Transwell assay. ^∗∗^*P* < 0.01.

To understand the underlying mechanism of exosomal miR-452-5p in M2 macrophage polarization, the targeted mRNA was predicted by TargetScan and starBase databases. Putting the results together, 226 mRNAs were found in common (Figure 6(a)). Next, we performed the Gene Ontology enrichment analysis and noticed the GO term negative regulation of metallopeptidase activity (Table S3). Among these genes, TIMP3 has been previously reported to regulate macrophage polarization [22, 23] and demonstrated to have a targeted binding site with miR-452-5p in our study. Dual luciferase assay displayed a decreased luciferase activity of TIMP3-WT group in PMA-induced THP-1 cells, suggesting the binding relationship (Figures 6(b) and 6(c)). In addition, treatment with exosomes derived from SNU-182 or Huh-7 cells also decreased the luciferase activity of TIMP3-WT group in PMA-induced THP-1 cells (Figure 6(d)). In PMA-induced THP-1 cells, TIMP3 mRNA and protein expression was reduced in miR-452-5p overexpressed cells (Figures 6(e) and 6(f)). Moreover, we cocultured PMA-induced THP-1 cells with miR-452-5p mimics and TIMP3 overexpression vector. The expressions of CD206, Arg-1, and IL-10 were increased after the overexpressing miR-452-5p, and reduced after further transfection with TIMP3 overexpression vector (Figure 6(g)). Similarly, TIMP3 reversed the effect of exosomes derived from SNU-182 or Huh-7 cells on the expression of M2 markers (Figure 6(h)). Furthermore, THP-1 cells were transfected with TIMP3 overexpression vector and cocultured with HCC cells-exo. As a result, the cell invasion and migration were promoted by HCC cells-exo and further reduced by overexpressing TIMP3 (Figure 6(i)). In summary, we suggested that TIMP3 could potentially play a role in M2 macrophage polarization that induced by HCC cells deserved exosomal miR-452-5p.

## 4. Discussion

So far, plenty of ncRNAs and proteins have been revealed as promising biomarkers of HCC, in which their dysregulations have been indicated to be related with tumor pathogenesis and more and more attention has been paid on miRNA involved regulatory mechanism in HCC [24, 25]. For instance, miR-452 has been identified as an oncogene in HCC patients and induced the stem-like features of HCC [26]. The study of Zheng et al. has revealed that miR-452-5p overexpression in HCC could accelerate tumorigenicity, metastasis, and self-renewal of tumor cells both *in vivo* and *in vitro*, while its silencing showed an opposite effect [27].

In our study, a high level of miR-452-5p was identified in HCC, and survival analyses also showed that a higher level of miR-452-5p was likely to indicate poorer prognosis of patients. Taken all these together, we suggested that miR-452-5p might be important in the regulatory mechanism of HCC progression. To demonstrate our hypothesis, we detected miR-452-5p in HCC cells and found a greatly promoted expression in HCC cells. Moreover, we observed a suppressive effect of miR-452-5p inhibition on the migration, invasion, and proliferation of HCC cells, whereas miR-452-5p overexpression showed a potentiated effect. As we got consistent results with previous HCC studies [15, 27], indicating that miR-452-5p overexpression could accelerate cell cycle and suppress cell apoptosis, the role of miR-452-5p in HCC as an oncogene can be expected.

The potential of exosomal miRNAs has been identified in recent tumor studies. Exosomes showed advantages over cell-free fluids like serum, which can selectively carry cell- and disease-specific noncoding RNAs and are more common in HCC patients [28]. In addition, exosomal noncoding RNAs has been proved to stay stable in serum after being secreted and present unique expression profiles, which help them become noninvasive biomarkers in HCC studies [29]. A study of Wang et al. detected miRNA expression of serum exosomes and found an obviously higher expression in subjects with HCC compared to normal controls [10]. Additionally, the effect of HCC-exo has also been indicated in the study of Wang et al., which carried lncRNA DLX6-AS1 to induce HCC cell migration [30]. Consistent with previous findings, we verified that miR-452-5p was encapsulated and protected against RNase and it might selectively reside in the exosome lumen secreted by HCC cells. Furthermore, we demonstrated that the inhibition of HCC cells deserved exosomal miR-452-5p could suppress cell migration and invasion and restrain tumor size, and M2 macrophage polarization might be involved.

HCC-derived exosomes can induce tumor-induced polarization of M2 macrophages, showing the important role of exosomes between HCC cells and macrophages [31]. As we know that the variations of microenvironment around tumor cells could influence the cellular transcriptional profiling [32]. Therefore, we investigated if HCC-exo could influence HCC cell proliferation and tumorigenesis by inducing the polarization of M2 macrophages. Several M1 and M2 macrophage makers including iNOS, IL-1*β*, CD206, Arg-1, and IL-10 were applied. We found that M2 macrophage marker proteins were significantly increased compared with the controls, which was consistent with our expectation. Moreover, by performing Transwell assay in transfected cells and coculturing HCC cells with HCC-exo-induced M2 macrophages, we found that the cocultivation of HCC cells and M2 macrophage treated with HCC-exo carrying miR-inhibitor could reduce migrated and invasive cells and decrease tumor size. Thus, HCC-exo-miR-452-5p was further demonstrated as a protumor factor in HCC by inducing M2 macrophages.

MiRNAs usually function in tumor diseases via regulating mRNAs, and the regulatory axis between miR-452-5p and mRNAs have been revealed in previous tumor studies. miR-452-5p has been found to target SMAD4 and influence cell biological behavior through the SMAD4/SMAD7 signaling pathway [33]. It has also been found to impede the process of EMT by the Bmi-1/AKT axis [34]. These results suggest that miR-452-5p can also play a series of roles by regulating its action targets. In this study, TIMP3 has been confirmed to be downregulated by miR-452-5p. In previous studies, TIMP3 was regarded as a tumor suppressor, since the knockout of TIMP3 can result in tumor aggressiveness and poor prognosis [35]. Moreover, the anticancer effect of TIMP3 has been revealed on HCC by promoting apoptosis and suppressing the amount of migrated and invasive cells [20]. Here, we demonstrated that TIMP3 was capable of normalizing the effect of HCC cells deserved exosomal miR-452-5p on M2 macrophage polarization and HCC cells metastasis.

## 5. Conclusion

Collectively, our study demonstrated that HCC cell exosome could carry miR-452-5p to macrophages and induce polarization of M2 macrophage, therefore promoting HCC cells invasion and migration and tumorigenesis, with TIMP3 served as an mRNA target. Although scientific demonstration for this study remains to perform in a larger cohort, our study may provide a promising pathway for understanding the underlying mechanism of HCC.

## Data Availability

The data used to support the findings of this study are included within the article.

## Supplementary Materials

Supplementary 1 Figure S1: miR-452-5p overexpression in HCC cells. (a) miR-452-5p overexpressed transfection models. (b, c) Cell viability and proliferation rate were detected by performing CCK-8 assay and EdU. (d) Apoptosis rate of miR-452-5p overexpressed. (e, f) Migration and invasion of miR-452-5p overexpressed cells were detected by Transwell assay. ^∗∗^*P* < 0.01.

Supplementary 2 Figure S2: induction of M0 macrophages. (a) Morphological identification of THP-1 cells with and without PMA induction. (b) Expression of CD68 in PMA-treated THP-1 cells. ^∗∗^*P* < 0.01.

Supplementary 3 Table S1: primers list.

Supplementary 4 Table S2: primary antibodies for western blot.

Supplementary 5 Table S3: Gene Ontology analysis of the 226 genes.
