# Supplementary figures and images for: Exosomal miR-452-5p Induce M2 Macrophage Polarization to Accelerate Hepatocellular Carcinoma Progression by Targeting TIMP3
Source: J Immunol Res. 2022 Sep 16;2022:1032106. doi: 10.1155/2022/1032106 (PMC9508462; doi:10.1155/2022/1032106)

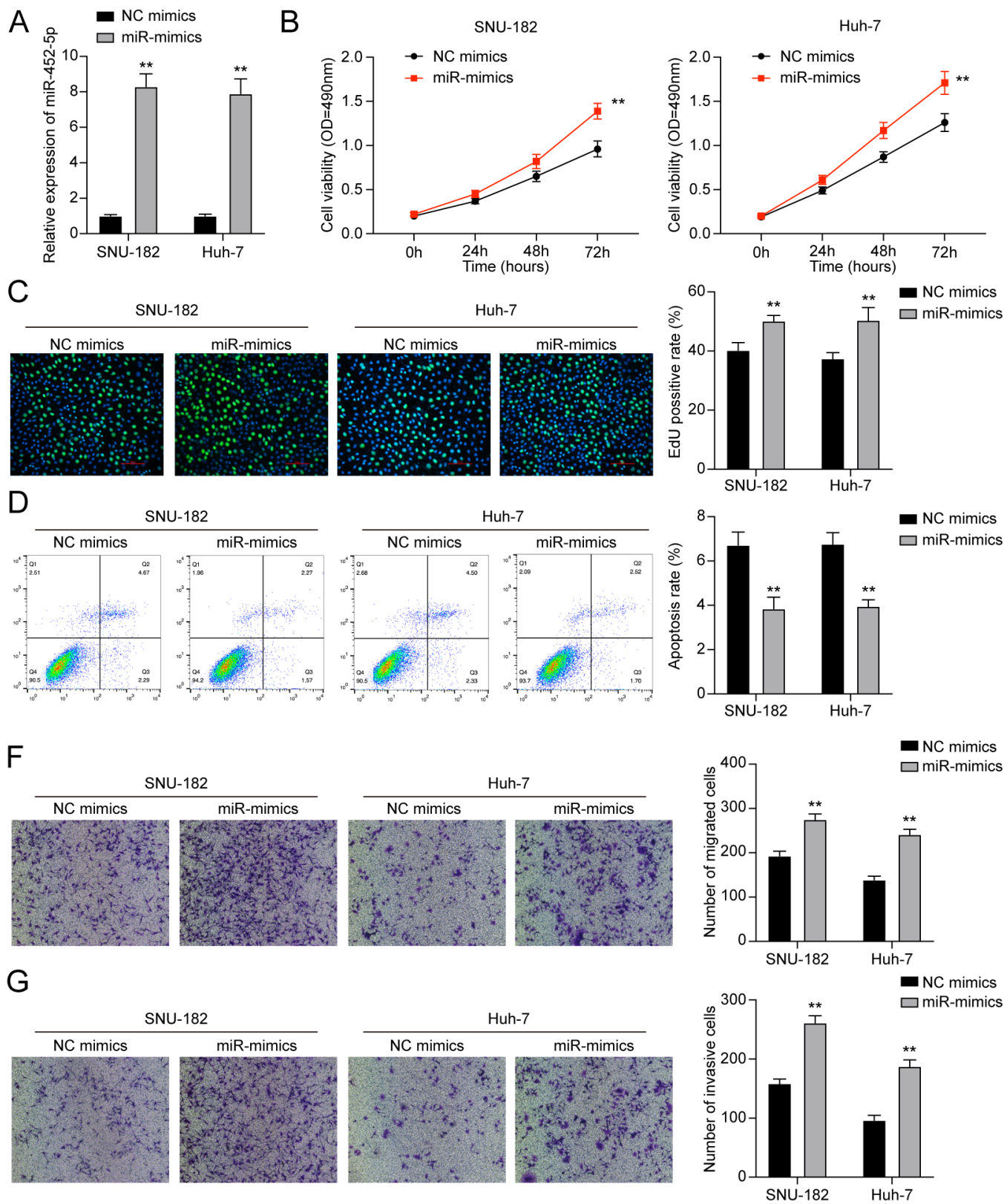

Supplement: Supplementary 1 — Figure S1: miR-452-5p overexpression in HCC cells. (a) miR-452-5p overexpressed transfection models. (b, c) Cell viability and proliferation rate were detected by performing CCK-8 assay and EdU. (d) Apoptosis rate of miR-452-5p overexpressed. (e, f) Migration and invasion of miR-452-5p overexpressed cells were detected by Transwell assay. ∗∗P < 0.01. [file 1032106.f1.pdf]

**A**

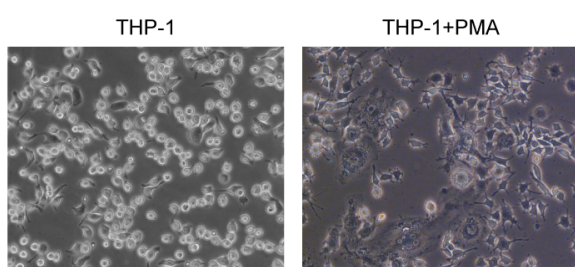

**B**

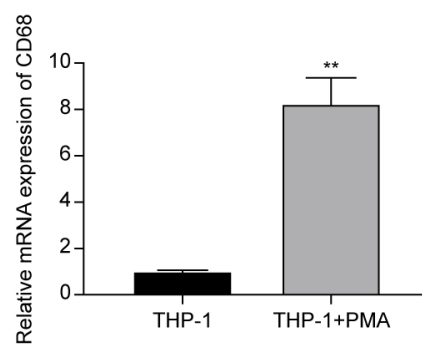

Supplement: Supplementary 2 — Figure S2: induction of M0 macrophages. (a) Morphological identification of THP-1 cells with and without PMA induction. (b) Expression of CD68 in PMA-treated THP-1 cells. ∗∗P < 0.01. [file 1032106.f2.pdf]
