# Supplementary material for: Exosomal miR-452-5p Induce M2 Macrophage Polarization to Accelerate Hepatocellular Carcinoma Progression by Targeting TIMP3
Source: J Immunol Res. 2022 Sep 16;2022:1032106. doi: 10.1155/2022/1032106 (PMC9508462; doi:10.1155/2022/1032106)
Supplement: Supplementary 3 — Table S1: primers list. [file 1032106.f3.pdf]

Table S1 Primers list

Gene	Forward Primer (5'-3')	Reverse Primer (5'-3')
miR-452-5p	AGCGCGAACTGTTTGCAGAGGA	ATCCAGTGCAGGGTCCGAGG
CD68	CTTCTCTCATTCCCCTATGGACA	GAAGGACACATTGTACTCCACC
CD206	GACGTGGCTGTGGATAAATAAC	CAGAAGACGCATGTAAAGCTAC
Arg-1	AGACCACAGTTTGGCAATTGG	AGGAGAATCCTGGCACATCG
IL-10	GTTGTTAAAGGAGTCCTTGCTG	TTCACAGGGAAGAAATCGATGA
iNOS	GCTCTACACCTCCAATGTGACC	CTGCCGAGATTTGAGCCTCATG
IL-1β	GCCAGTGAAATGATGGCTATT	AGGAGCACTTCATCTGTTTAGG
TIMP3	GTGCAACTTCGTGGAGAGGT	CAGGTAGCAGGACTTGATCTTG
GAPDH	AACGGATTTGGTCGTATTGG	TTGATTTTGGAGGGATCTCG
U6	CTCGCTTCGGCAGCACA	AACGCTTCACGAATTTGCGT
