# Supplementary material for: Exosomal miR-452-5p Induce M2 Macrophage Polarization to Accelerate Hepatocellular Carcinoma Progression by Targeting TIMP3
Source: J Immunol Res. 2022 Sep 16;2022:1032106. doi: 10.1155/2022/1032106 (PMC9508462; doi:10.1155/2022/1032106)
Supplement: Supplementary 4 — Table S2: primary antibodies for western blot. [file 1032106.f4.pdf]

Table S2 Primary antibodies for western blot

Antibody	Concentration	Specificity	Catalog number	Company
TSG101	1/2000	Rabbit monoclonal	ab125011	Abcam
CD9	1/2000	Rabbit monoclonal	13403	Cell Signaling Technology
Alix	1/2500	Rabbit monoclonal	92880	Cell Signaling Technology
TIMP3	1/2000	Rabbit monoclonal	5673	Cell Signaling Technology
GAPDH	1/2500	Rabbit polyclonal	ab9485	Abcam
