# Supplementary material for: Exosomal miR-452-5p Induce M2 Macrophage Polarization to Accelerate Hepatocellular Carcinoma Progression by Targeting TIMP3
Source: J Immunol Res. 2022 Sep 16;2022:1032106. doi: 10.1155/2022/1032106 (PMC9508462; doi:10.1155/2022/1032106)
Supplement: Supplementary 5 — Table S3: Gene Ontology analysis of the 226 genes. [file 1032106.f5.pdf]

Table S3 Gene Ontologies analysis of the 226 genes

Term	P-value
**GO Biological Process**	
regulation of transcription by RNA polymerase II (GO:0006357)	1.83E-07
regulation of transcription, DNA-templated (GO:0006355)	3.13E-07
positive regulation of transcription, DNA-templated (GO:0045893)	1.43E-06
positive regulation of transcription by RNA polymerase II (GO:0045944)	1.32E-05
regulation of calcium ion transport (GO:0051924)	4.83E-05
negative regulation of calcium ion transport (GO:0051926)	5.39E-05
negative regulation of transcription, DNA-templated (GO:0045892)	7.50E-05
negative regulation of gene expression (GO:0010629)	8.10E-05
cellular protein modification process (GO:0006464)	1.01E-04
negative regulation of transcription by RNA polymerase II (GO:0000122)	1.04E-04
cellular response to decreased oxygen levels (GO:0036294)	1.29E-04
nervous system development (GO:0007399)	1.82E-04
transcription by RNA polymerase II (GO:0006366)	2.99E-04
membrane lipid biosynthetic process (GO:0046467)	4.96E-04
axon guidance (GO:0007411)	5.20E-04
endoderm development (GO:0007492)	5.86E-04
phosphorylation (GO:0016310)	6.60E-04
positive regulation of nucleic acid-templated transcription (GO:1903508)	7.41E-04
tail-anchored membrane protein insertion into ER membrane (GO:0071816)	8.61E-04
negative regulation of insulin secretion (GO:0046676)	8.61E-04
negative regulation of histone H3-K27 methylation (GO:0061086)	0.001243044
negative regulation of metallopeptidase activity (GO:1905049)	0.001243044
regulation of ubiquitin-dependent protein catabolic process (GO:2000058)	0.001269089
**GO Molecular Function**	
transcription coregulator binding (GO:0001221)	3.25E-04
nuclear import signal receptor activity (GO:0061608)	7.15E-04
double-stranded DNA binding (GO:0003690)	0.001218734
RNA polymerase II cis-regulatory region sequence-specific DNA binding (GO:00(	0.001308305
transcription coactivator binding (GO:0001223)	0.001408196
cyclin-dependent protein serine/threonine kinase regulator activity (GO:0016538)	0.001512321
nuclear localization sequence binding (GO:0008139)	0.002418378
calcium-dependent protein serine/threonine phosphatase activity (GO:0004723)	0.002571685
DNA-binding transcription factor binding (GO:0140297)	0.002585174
cis-regulatory region sequence-specific DNA binding (GO:0000987)	0.002781721
sequence-specific DNA binding (GO:0043565)	0.002890471
transcription cis-regulatory region binding (GO:0000976)	0.004002936
histone deacetylase binding (GO:0042826)	0.004093295
transcription corepressor binding (GO:0001222)	0.004188619
double-stranded telomeric DNA binding (GO:0003691)	0.004343344
RNA binding (GO:0003723)	0.004842504
RNA polymerase II transcription regulatory region sequence-specific DNA bindin[illegible]	0.005955587
**GO Cellular Component**	
intracellular membrane-bounded organelle (GO:0043231)	4.38E-08
nucleus (GO:0005634)	3.24E-07

Adjusted P-v	Odds Ratio	Combined Sc	Genes
2.80E-04	2.444636549	37.92336197	ARF4;CCNT2;THRB;ZBTB20;HMGB3;TCF20;RORA;
2.80E-04	2.397076097	35.89742494	TENM1;CDKN1B;CCNT2;HP1BP3;ZBTB20;HMGB3;[illegible]
8.54E-04	2.769056094	37.25778698	ARF4;CCNT2;THRB;TCF20;RORA;HOXC13;RPS6KA
0.005918659	2.784535147	31.27490911	ARF4;CCNT2;THRB;TCF20;RORA;SUPT6H;RPS6KA
0.016040187	10.34370629	102.8004246	STIM2;CACNA2D1;STC1;ATP2B1;CAMK2G;ADRA2
0.016040187	23.73453453	233.2912981	STC1;SLC30A1;TMBIM6;ADRA2A
0.018082871	2.540251074	24.12689685	CDKN1B;THRB;BMI1;PHF6;PHF21A;PURA;HMBOX
0.018082871	3.844675388	36.22315554	MEF2C;SAMD4A;ATP2B1;BMI1;CRKL;VEGFA;AEB
0.018661263	2.443193193	22.48446632	ARF4;DYRK2;SDC2;DCUN1D1;PTPRJ;LAMC1;PPP2[illegible]
0.018661263	2.794186455	25.61434588	SMAD2;KLF10;MEF2C;DR1;KLF12;THRB;BMI1;PHF
0.020952144	8.532900433	76.4199102	NPEPPS;HP1BP3;PGK1;RORA;HIPK2;VEGFA
0.027160768	3.182925224	27.40297638	ROBO2;TENM1;MEF2C;RYK;BDNF;TMOD2;DYRK1
0.041157134	3.543998058	28.75494473	CCNT2;THRB;COPS2;LHX1;TRIM24;RORA;ESRRG;[illegible]
0.061968003	8.418423973	64.05255104	CERS6;SAMD8;SPTLC2;VAPB;ST3GAL2
0.061968003	4.185947076	31.65153825	ROBO2;KLF7;RYK;BDNF;LHX1;CHN1;DAG1;MAPK
0.065444629	22.15470852	164.8810046	LHX1;LAMC1;SOX7
0.069335988	3.057478376	22.39257833	DYRK2;RYK;DYRK1A;PRPF4B;HIPK2;PPP3CB;COP
0.073609893	2.763052286	19.9130349	SMAD2;MEF2C;DYRK1A;RORA;ESRRG;HOXC13;I[illegible]
0.076951282	18.98782832	133.9998386	CAMLG;MMGT1;EMC8
0.076951282	18.98782832	133.9998386	KLF7;KCNB1;ADRA2A
0.098602694	58.8422619	393.6660514	MTF2;SUPT6H
0.098602694	58.8422619	393.6660514	TIMP2;TIMP3
0.098602694	9.357989569	62.41269966	RNF139;UBE3A;RAD23B;HIPK2
0.070070865	9.297699849	74.66853137	CCNT2;THRB;MTF2;RORA;ZNF644
0.070070865	20.44946533	148.1146665	KPNA4;RANBP6;TNPO1
0.070070865	2.455586917	16.47684744	SMAD2;KLF10;FOXB1;MEF2C;KLF12;THRB;ZBTB2
0.070070865	2.063747101	13.7012648	THRB;ZBTB20;RORA;HOXC13;CHD2;HOXA3;TRIM
0.070070865	15.63466104	102.6485165	CCNT2;THRB;RORA
0.070070865	8.889189189	57.72737097	CCNT2;CDKN1B;CNPPD1;CCNO
0.073050089	12.65406791	76.23643531	KPNA4;RANBP6;TNPO1
0.073050089	35.30178571	210.5113973	PPP3CB;CAMK2G
0.073050089	3.591559633	21.39837729	SMAD2;RERE;PURA;MEF2C;HOXC13;GABPA;HIPK
0.073050089	1.969531353	11.59007255	SMAD2;KLF10;FOXB1;MEF2C;KLF12;THRB;ZBTB2
0.073050089	2.249691422	13.15245142	KLF10;FOXB1;MEF2C;KLF12;THRB;ZBTB20;RORA;
0.080496649	2.374766355	13.11043731	SMAD2;KLF10;MEF2C;DR1;ZBTB20;RORA;HOXC1[illegible]
0.080496649	5.061188811	27.82846589	MEF2C;BRMS1L;PKN2;PHF6;DACT1
0.080496649	10.21800621	55.94751019	MTF2;RORA;ZNF644
0.080496649	25.2130102	137.1363519	PURA;HMBOX1
0.084138514	1.809865134	9.647166215	RBM28;PPP1R10;CELF2;IREB2;HMGB3;TCF20;CHD[illegible]
0.09739136	1.798447112	9.214209876	THRB;ZBTB20;RORA;HOXC13;CHD2;PURA;HOXA[illegible]
7.48E-06	2.126989494	36.04067415	RERE;TENM1;CDKN1B;CCNT2;DYRK2;THRB;PPP1
2.77E-05	2.066927017	30.88524164	RERE;TENM1;CDKN1B;CCNT2;DYRK2;THRB;PPP1

HOXC13;BMI1;CHD2;PHF6;RPS6KA3;PPP3CB;HOXA3;ZNF644;TEAD1;RNF111;GABPA;SO[illegible]
TCF20;RORA;HOXC13;CHD2;PHF6;ELAVL2;RPS6KA3;PPP3CB;HOXA3;ZNF644;SOX7;TEA[illegible]
[illegible]3;PPP3CB;SERTAD2;E2F3;TEAD1;RNF111;GABPA;WNT4;SMAD2;ACTR3;KLF10;ZNF462;[illegible]
[illegible]3;PPP3CB;E2F3;TEAD1;RNF111;GABPA;SMAD2;ACTR3;KLF10;ZNF462;MEF2C;DR1;SMAR[illegible]
[illegible]A

[illegible]1;ZNF148;SOX7;WNT4;DACT1;SMAD2;KLF10;MEF2C;DR1;KLF12;FOXP2;VEGFA;KLF7;LH[illegible]
[illegible]P2;RNF139;MTF2;TMBIM6;IGF2BP2;ZNF148;WNT4
[illegible]CA;PPP3CB;CAND1;TLK1;MAPK1;ST3GAL2;SKP1;SOCS5;RYK;DYRK1A;PRPF4B;FBXO11;[illegible]
[illegible]6;FOXP2;PHF21A;VEGFA;PURA;KLF7;HMBOX1;BRMS1L;MTF2;ZNF148;MXD1;FRK;DAC[illegible]

[illegible]A;INHBA;VEGFA;RPS6KA3;NAV3;LHX1;IGF2BP2;TEAD1;DACT1
E2F3;INTS8;TEAD1;SUPT6H;SOX5

[illegible]1;VEGFA

[illegible]S2;PGK1;TLK1;MAPK1;PKN2;XYLB;FRK
[illegible]HBA;RGMB;HIPK2;PPP3CB;LHX1;NAA15;TEAD1;RNF111;WNT4

[illegible]0;HMGB3;ESRRG;HOXC13;PURA;HMBOX1;LHX1;E2F3;SOX7;TEAD1;GABPA
[illegible]24;E2F3;ZNF644;ZNF148;SOX7;TEAD1;GABPA;ZKSCAN1;SOX5;ZBTB7C;SMAD2;KLF10;F[illegible]

[illegible]2;DACT1
[illegible]0;RORA;ESRRG;HOXC13;CHD2;FOXP2;KLF7;HOXA3;E2F3;ZNF644;ZNF148;MXD1;SOX7;T[illegible]
[illegible];ESRRG;HOXC13;FOXP2;HMBOX1;LHX1;E2F3;ZNF148;SOX7;TEAD1;GABPA
[illegible]3;PURA;LHX1;E2F3;SOX7;TEAD1;GABPA;SOX5

[illegible]2;PHF6;BZW1;SUPT6H;ELAVL2;CRKL;PURA;IGF2BP2;TNPO1;SF3B1;AGFG1;SPATS2;SAM[illegible]
[illegible]3;E2F3;ZNF644;ZNF148;SOX7;TEAD1;GABPA;ZKSCAN1;SOX5;ZBTB7C;SMAD2;KLF10;FO[illegible]

R10;CELF2;HP1BP3;ZBTB20;TCF20;RORA;MSANTD4;ZC3H6;BMI1;CHD2;PHF6;TNFSF13B;[illegible]
R10;CELF2;HP1BP3;ZBTB20;TCF20;RORA;MSANTD4;ZC3H6;BMI1;CHD2;PHF6;SUMO3;H[illegible]

K5;DACT1;ACTR3;KLF10;MEF2C;KLF12;ZFP91;FOXP2;AEBP2;MTF2;NCOA7;ITGA6;PHF21
D1;RNF111;GABPA;SOX5;KLF10;MEF2C;KLF12;DYRK1A;ZFP91;RGMB;FOXP2;AEBP2;MTF
IEF2C;DR1;SMARCA5;DYRK1A;ESRRG;INHBA;RGMB;VEGFA;HIPK2;KLF7;LHX1;MTF2;N
CA5;INHBA;VEGFA;HIPK2;KLF7;MTF2;NCOA7;ITGA6

X1;BRMS1L;MTF2;MXD1;RCOR1;FRK

DUSP6;RAB11A;FBXW2;HIPK2;COPS2;PKN2;KBTBD7;FRK
'1

OXB1;MEF2C;KLF12;ESRRG;FOXP2;KLF7;MXD1

EAD1;GABPA;ZKSCAN1;SOX5;ZBTB7C

D4A;PRPF4B;DHX40;XPOT;CANX;PKN2;MATR3;PABPC1;NAA15
XB1;MEF2C;KLF12;ESRRG;FOXP2;KLF7;LHX1;MXD1

MAP1LC3B;SUMO3;NCS1;DAG1;HOXA3;TRIM24;TIMP3;TLK1;KPNA4;ARIH1;ZNF644;TNP
OXA3;TRIM24;TIMP3;TLK1;KPNA4;ARIH1;ZNF644;TNPO1;SOX7;CNPPD1;RNF111;GABPA;S

A;PURA;HMBOX1;E2F3;ZNF148;ZKSCAN1;ZBTB7C;SMAD2;FOXB1;ZNF462;DR1;SMARCA:
'2;NCOA7;PPP2CA;PURA;HMBOX1;ATXN1L;E2F3;ZNF148;ZKSCAN1;ZBTB7C;WNT4;SMAI
COA7;ITGA6;NAA15

O1;SOX7;CNPPD1;RNF111;GABPA;SOX5;ANKS1B;AGFG1;SKP1;DACT1;ACTR3;KLF10;ME
SOX5;SKP1;DACT1;ACTR3;KLF10;MEF2C;REXO1;DYRK1A;ZFP91;PRPF4B;FBXO11;RAD23

5;ESRRG;INHBA;VEGFA;HIPK2;KLF7;LHX1;BRMS1L;MXD1;RCOR1;FRK
D2;FOXB1;SMARCA5;ESRRG;INHBA;VEGFA;HIPK2;KLF7;LHX1;NAA15;MXD1;RCOR1

F2C;REXO1;DYRK1A;ZFP91;PRPF4B;FBXO11;RAD23B;FOXP2;PISD;RAB30;RNF169;MTF2;
B;FOXP2;PISD;RNF169;MTF2;HAT1;NCOA7;MATR3;TMBIM6;PABPC1;CCNO;UBA6;MGRN

HAT1;RAPGEF1;NCOA7;MATR3;TMBIM6;PABPC1;CCNO;UBA6;MGRN1;DCUN1D1;STC1;[illegible]
[illegible]1;DCUN1D1;STC1;NREP;OAZ2;GMCL1;MIPOL1;PPP2CA;PURA;HMBOX1;CAND1;SERTAD

[illegible]BHD5;NREP;OAZ2;GMCL1;MIPOL1;PPP2CA;PURA;HMBOX1;CAND1;SERTAD2;ATXN1L;[illegible]
[illegible]2;ATXN1L;MAPK1;E2F3;IGF2BP2;SF3B1;SMAD2;FOXB1;ZNF462;RYK;SMARCA5;RANBP6

MAPK1;E2F3;IGF2BP2;SF3B1;SMAD2;FOXB1;ZNF462;RYK;YIPF4;SMARCA5;ATP2B1;RAN[illegible]
;NAP1L5;HIPK2;KLF7;LHX1;PKN2;NAA15;RCOR1;FRK

BP6;RAB11A;NAP1L5;HIPK2;KLF7;LHX1;PKN2;NAA15;RCOR1;FRK
